# Dynamic Imaging of Marrow-Resident Granulocytes Interacting with Human Mesenchymal Stem Cells upon Systemic Lipopolysaccharide Challenge

**DOI:** 10.1155/2013/656839

**Published:** 2013-03-27

**Authors:** Jay T. Myers, Deborah S. Barkauskas, Alex Y. Huang

**Affiliations:** ^1^Department of Pediatrics, Division of Pediatric Hematology/Oncology, Case Western Reserve University School of Medicine, Cleveland, OH 44106-7288, USA; ^2^Department of Pathology, Case Western Reserve University School of Medicine, Cleveland, OH 44106-7288, USA

## Abstract

Human mesenchymal stem cells (hMSCs) have gained intense research interest due to their immune-modulatory, tissue differentiating, and homing properties to sites of inflammation. Despite evidence demonstrating the biodistribution of infused hMSCs in target organs using static fluorescence imaging or whole-body imaging techniques, surprisingly little is known about how hMSCs behave dynamically within host tissues on a single-cell level *in vivo*. Here, we infused fluorescently labeled clinical-grade hMSCs into immune-competent mice in which neutrophils and monocytes express a second fluorescent marker under the lysozyme M (LysM) promoter. Using intravital two-photon microscopy (TPM), we were able for the first time to capture dynamic interactions between hMSCs and LysM^+^ granulocytes in the calvarium bone marrow of recipient mice during systemic LPS challenge in real time. Interestingly, many of the infused hMSCs remained intact despite repeated cellular contacts with host neutrophils. However, we were able to observe the destruction and subsequent phagocytosis of some hMSCs by surrounding granulocytes. Thus, our imaging platform provides opportunities to gain insight into the biology and therapeutic mechanisms of hMSCs *in vivo* at a single-cell level within live hosts.

## 1. Introduction

Human mesenchymal stem cells (hMSCs) are self-renewing precursor cells capable of differentiating into bone, adipose tissue, cartilage, and stromal cells of the bone marrow depending on the stimuli [1]. Available data indicate that hMSCs are pericytes whose pleiotropic nature allows them to sense and respond to inflammatory processes in the microenvironment [2]. Although hMSCs are found at low frequency *in vivo *in a variety of adult tissues including bone marrow, muscle, fat, and dermis, they can be expanded to large numbers under appropriate culture conditions. For this reason, hMSCs have been applied therapeutically in rapidly expanding clinical investigations including more than 200 currently active clinical trials worldwide [1, 3–6]. A wide range of diseases including diabetes, atherosclerosis, multiple sclerosis, systemic lupus, Crohn's disease, myocardial infarction, stroke, Parkinson's disease, bone and cartilage repair, wound healing, and graft-versus-host disease [1, 3] have been treated using hMSCs as a cellular therapy. These clinical trials aimed to explore the therapeutic potential of hMSCs with regard to their immune-modulatory properties, tissue regenerative capacity, graft enhancement, tissue protection, and repair capabilities. Similarly, hMSCs have been applied *in vivo* for their efficacy in a variety of human disease models in immune-competent mice including skin and spinal cord repair [7], Huntington's disease, [8], other demyelinating diseases [9], and graft-versus-host disease [10]. 

Despite intense research interest and active clinical applications of hMSCs, there has been some controversy and little evidence regarding the biodistribution and actual cellular behavior of hMSCs upon infusion *in vivo*. Investigators have utilized a variety of whole-body imaging and static histological analyses to track the presence and possible local function of hMSCs in diseased and normal anatomical sites [11]. These tracking modalities include magnetic resonance imaging [12, 13], near-infrared whole-body *in vivo* imaging system [14], chromium^51^ (Cr-51) tracking [15], and bioluminescence and static fluorescence microscopy [16–21]. Recently, Eggenhofer et al. traced the fate of Cr-51 labeled syngeneic MSCs that had been infused intravenously into C57BL/6 mice and showed that viable MSCs could only be recovered in the lungs up to 24 hours after infusion [22]. Based on these data, the authors concluded that MSCs are rather short-lived after intravenous infusion, that viable MSCs were trapped within the lung tissue with some cellular debris transported to and cleared in the liver, and that infused MSCs must exert their immune-modulatory and regenerative effects via a third-party cell type. 

All of the studies mentioned above were useful in demonstrating that, under inflammatory insults or tissue injury, infused hMSCs accumulate at disease sites, thus supporting the hypothesis of their *in vivo *survival and homing capacity to target organs. However, whole-organ or whole-mouse tracking studies lacked the single-cell resolution within the intact tissue microenvironment to fully explore the behavior of infused hMSCs. On the other hand, high-resolution detection of fixed tissue analyses was devoid of the dynamic information regarding cellular migrations and interactions, which are hallmarks of essential immune cell functions *in vivo*. What would be more informative is the study of individual hMSC interaction dynamics within undisrupted live tissue microenvironment in real time. 

In our current study, we utilized intravital TPM to monitor intravenously infused, fluorescently labeled hMSCs in the bone marrow of immune competent mice in which host myeloid cells expressing lysozyme M (LysM)—principally neutrophils and monocytes—can be concurrently tracked at the single-cell level. By observing in high-definition the interaction dynamics between hMSCs and host innate immune cells before and after a systemic LPS challenge, we provide the first glimpse into how hMSCs behave locally with surrounding immune cells in the native murine bone marrow environment, thereby directly refuting the claims of a previous study which argues that since viable MSCs do not go past the lungs, the immune-modulatory and regenerative effects of infused MSC must therefore be mediated via other cell types. Furthermore, we also captured *in vivo* the active process of hMSC destruction by LysM-expressing cells in the bone marrow.

## 2. Materials and Methods

### 2.1. Mice

C57BL/6 mice (stock #664) were obtained from Jackson Laboratory (Bar Harbor, ME, USA). C57BL/6 mice containing GFP inserted into the *lysozyme M* locus (*LysM* ^+/*GFP*^) were obtained from Dr. Thomas Graf [23]. Eight-to-twelve-week-old mice were used for these experiments. Animals were housed, bred, and handled in the Animal Resource Center facilities at Case Western Reserve University according to the approved protocols. Similarly, all animal experiments were executed with strict adherence to active experimental animal protocols approved by Case Western Reserve University Institutional Animal Care and Use Committee.

### 2.2. Cell Labeling and Injections

Clinical-grade marrow-derived hMSCs were obtained from the Hematopoietic Stem Cell Core Facility in the Case Comprehensive Cancer Center at Case Western Reserve University where they had been expanded and characterized to possess renewal and tri-lineage differentiation potential (osteogenic, chondrogenic, and adipogenic) [24–28]. We refer to these cells as hMSCs rather than hMSPCs (human mesenchymal stem and progenitor cells) (1) since these hMSCs have been demonstrated to enrich stem cell activity as part of the routine protocol in the Hematopoietic Stem Cell Facility at the Case Comprehensive Cancer Center. Briefly, aliquots of frozen hMSCs were thawed and washed in pre-warmed complete media (*α*MEM supplemented with L-glutamine, pen/strep, Fungizone, and 10% FCS). They were then washed twice with PBS and subsequently labeled with either 5 *μ*M CellTracker Red CMTPX (Invitrogen) or 2.5 *μ*M CellTracker Orange CMTMR (CTO; Invitrogen) at room temperature in PBS for 15 minutes. Two-to-four-fold volumes of PBS with 1% FBS were added to quench the labeling reaction and the cells were washed twice with PBS. Labeled cells were then injected *i.v*. at various concentrations. Where indicated, mice were also injected *i.v.* with 100 ng of *S. enterica* LPS (Sigma).

### 2.3. Mouse Surgery and Preparation for Intravital Imaging

 Intravital TPM of the calvarium was performed as previously described [29]. Briefly, mice were anesthetized with nebulized isoflurane (2% induction, 1.5% maintenance) in 30% O_2_/70% air and placed in a stereotactic holder. The hair on top of the skull was clipped and the remaining hair removed with Nair hair remover. The skin was then excised, and dental acrylic was used to create a trough on top of the calvarium to maintain the water column to facilitate imaging. The animal body temperatures were monitored and maintained between 36.5 and 38°C using a combination of a temperature-controlled environmental chamber, heating pads, and a rectal probe throughout the entire mouse preparation and imaging session. Breathing rate and animal responsiveness were used to ensure adequate levels of anesthesia. Respiratory rate was maintained at ~60–100 breaths per minute and animal responsiveness was assessed by foot and tail pinch. Ten to 30 minutes prior to imaging, mice were injected *i.v.* with either 100 *μ*L of 2.5 mg/mL FITC-dextran (Sigma) or 100 *μ*L of 0.2 *μ*M QTracker-655 (Invitrogen) to allow blood vessel visualization as indicated. 

### 2.4. TPM Equipment and Data Acquisition

Upon completion of the tissue preparation for intravital imaging, the entire mouse imaging assembly, including the stereotactic holder, was placed on the microscope stage that was enclosed within a custom-made temperature-controlled environmental chamber. The tissues were imaged using a Leica SP5 fitted with a DM6000 stage, a 20X water immersion lens (N.A. 1.0; Leica HCX-APO-L), and a 16W Ti/Sapphire IR laser (Chameleon, Coherent) tuned to excitation wavelengths between 800 nm and 860 nm. Imaging planes (776 × 776 *μ*m) collected at 5 *μ*m *z* steps were repeated at 30-second intervals for up to 3 hours to yield *xyzt* datasets collected through a four-channel nondescanned external detector using a filter set separating ≤495 nm, 500–550 nm, 565–605 nm, and 625–675 nm emission spectra. The raw imaging data set was then used for the processing and analysis using Imaris software (BitPlane, Inc.) as described below. 

### 2.5. Immunofluorescence Histology

After completion of intravital bone marrow imaging, hMSC-infused *LysM* ^+/*GFP*^mice were sacrificed by CO_2_ asphyxiation. Lungs, liver, lymph node, and spleen were harvested and fixed in 2% PFA at 4°C. High-resolution *xyz* imaging stacks of the fixed, unsectioned samples were collected using the Leica SP5/two-photon imaging equipment as described above. Image stacks were analyzed using Imaris software (BitPlane, Inc.) as described below. 

### 2.6. Image Analysis

 High-resolution fluorescent 4D imaging data sets collected from intravital TPM experiments were analyzed using the Imaris software (BitPlane, Inc.). A typical imaging volume of 776 × 776 × 150 *μ*m^3^ was analyzed. We utilized channel subtraction algorithms available in the Imaris Software in order to determine the localization of hMSC-CTO signal in relation to the LysM-GFP signal in the bone marrow. In short, a new CTO channel was created using the Matlab Channel Arithmatics Function in Imaris in which the CTO signals (channel 3, AKA ch3) which overlap the GFP signal (ch2) were removed by subtraction: ch3-ch2. This step subtracts the fluorescence of the GFP channel from the CTO channel at the pixel level. Cell identification and tracking were performed using the Spots Analysis Function in Imaris with the cell diameter set at 15 *μ*m. The *xyzt* positional data for both the hMSCs and LysM^+^ cells was exported to Matlab for further analysis of interaction frequency between hMSCs and LysM^+^ neutrophils. We defined neutrophil interaction with the hMSC by parsing the neutrophils whose center of mass was within a 20 *μ*m radius from that of a hMSC, compared the number of neutrophils that satisfied this positional requirement to the total cell number in the image field, and averaged over 30-minute intervals to derive the time average % of cellular interactions. To determine the directional migration of the neutrophils in relation to the hMSCs, we compared the angular components of individual instantaneous velocity of the neutrophils with 25 *μ*m radius to the hMSC and compared them to the vector angle formed between the centers of the neutrophil and the hMSC. We identified any neutrophils that were either moving towards (<95°) or away (>95° and <180°) from the hMSC and calculated a “cell flux index” defined as: (number of cells migrating towards-number of cells migrating away)/total cells within the 25 *μ*m radius of the hMSC. The cells designated as moving towards the hMSC include those that possess a tangential directional angle (at 90°), with an absolute angular limit value of 95°.

## 3. Results

**Figure 1:**
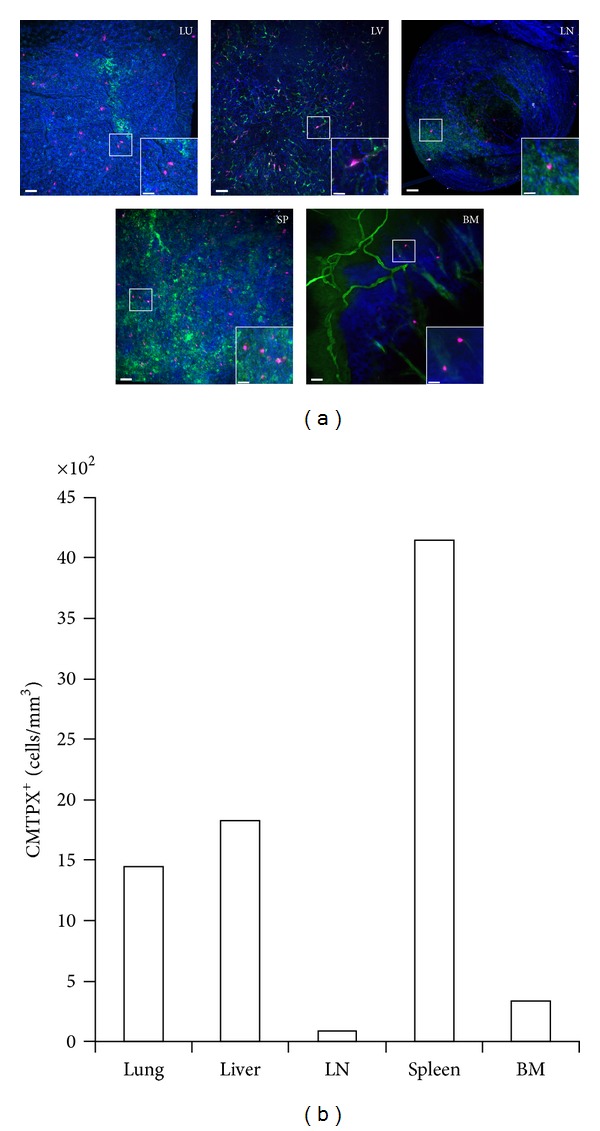
Distribution of CMTPX-labeled hMSCs 24 hours after i.v. injection into a C57BL/6 mouse.FITC-dextran was injected 15–30 minutes before intravital TPM imaging to highlight the vasculature. The calvarium bone marrow (BM) was imaged immediately and all other tissues were removed and fixed overnight in 2% PFA for whole-organ TPM imaging the next day. (a) Representative high-resolution TPM images of lung (LU), liver (LV), lymph node (LN), spleen (SP), and bone marrow (BM) are shown. Blue: collagen (second-harmonics); Green: FITC-dextran vessel dye; Magenta: infused hMSCs. Outset scale bars = 50 *μ*m; inset scale bars = 20 *μ*m. (b) Numbers of CMTPX^+^ hMSC cells per mm^3^ of imaged tissue were enumerated.

To test the biodistribution and tissue homing potential of the hMSCs in immune-competent mice, we fluorescently labeled 4 × 10^6^ hMSCs and administered them intravenously into C57BL/6 recipient mice. One day later, these mice were then subjected to both intravital TPM imaging of the bone marrow through intact calvarium, and static, fixed whole-organ TPM imaging of the lung, liver, lymph node, and spleen (Figure 1(a)). As expected from other published reports, we found a large number of the infused hMSCs in the lungs, liver and spleen, with 1443, 1825, and 4142 cells per mm^3^ of imaged tissue, respectively. Interestingly, we were able to easily detect fewer numbers of hMSCs in the lymph node and the bone marrow, with 90 and 337 cells per mm^3^ of imaged tissues, respectively (Figure 1(b)). 

**Figure 2:**
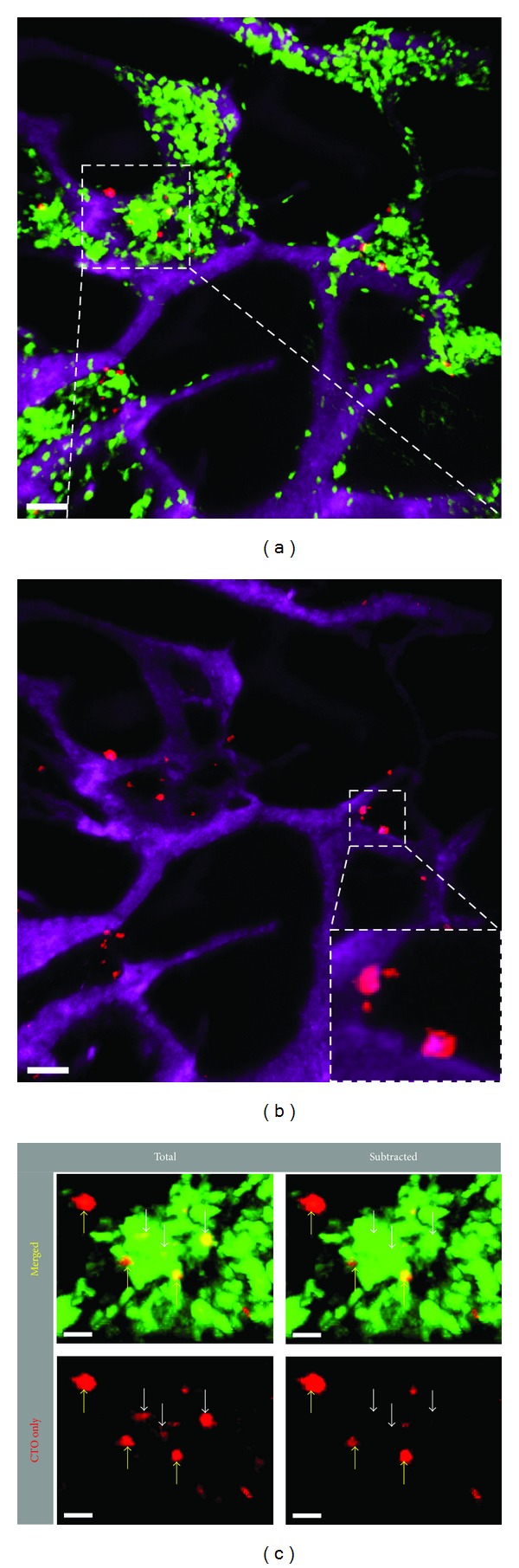
Distribution of CTO-labeled hMSCs in the calvarium of a *LysM* ^+/*GFP*^ mouse. A total of 8 × 10^6^ CTO-labeled hMSCs (red) were injected *i.v.* into a *LysM* ^+/*GFP*^ recipient mouse containing LysM^+^ (green) neutrophils and monocytes. Intravital TPM was performed through intact calvarium of live recipient mice 20 hours after *i.v.* injection of hMSCs. (a) Distribution of labeled hMSCs in the calvarium bone marrow within the general vicinity of bone marrow-resident LysM^+^ myeloid-lineage cells. Vessels were highlighted with Qtracker-655 (magenta). Scale bar = 50 *μ*m. (b) Same image as in (a) after channel subtraction to remove CTO signal inclusions inside of the LysM^+^ GFP signals, showing close association of the hMSCs with the vessels (inset). (c) Zoomed-in view of the inset in (a), showing that many hMSCs (red) remain intact in the bone marrow and do not colocalize with the GFP signal (yellow arrows). However, a small number of LysM^+^ granulocytes can be seen to harbor CTO^+^ inclusions inside the cell body (white arrows). Channel subtraction of the micrograph in the left column removed CTO signal that resided within LysM^+^ cell body, further supporting the presence of CTO signal inside a few LysM^+^ cells (right column; white arrows). Scale bars = 20 *μ*m.

Even though we could easily visualize individual CMTPX^+^ signals in various anatomical sites following hMSC infusion, these initial *in vivo *whole-organ tracking experiments of fluorescently labeled hMSCs were against a backdrop of the dark background in the imaging fields which are known to be packed full of a myriad of nonfluorescent host cells. In particular, from the first set of experiments, we could not formally distinguish whether the visualized fluorescent signals were coming from intact hMSCs, or from phagocytes such as neutrophils or monocytes that had phagocytosed dead hMSC debris that arrived at the liver, lymphoid organs, or the bone marrow. Indeed, a recent report indicates that, at least in the murine system, live mouse MSCs that had been administered intravenously could only be found in the lungs, whereas only MSC debris was found in the liver and spleen [22]. In order to rigorously test this, we employed high-resolution dynamic TPM imaging to the bone marrow cavity underneath the calvarium. Furthermore, we chose as recipients the *LysM* ^+/*GFP*^ mice in which the gene encoding the green fluorescent protein (GFP) replaced the *LysM *locus encoding lysozyme M, an enzyme that is highly expressed in granulocytes such as neutrophils and, to a lesser extent, monocytes [23]. Using this combination of fluorescent probes, we set out to observe the cellular integrity and interactions between labeled hMSCs with bone-marrow-resident neutrophils and monocytes (“granulocytes”). We fluorescently tagged 8 × 10^6^ hMSCs with CTO and injected them *i.v.* into a naive *LysM* ^+/*GFP*^ mouse, then performed intravital TPM imaging of the bone marrow cavity in the mouse calvarium a day later. As shown in Figure 1, multiple hMSCs could be visualized to colocalize in the same general vicinity of the bone marrow with the LysM^+^ granulocytes, with most hMSCs in close proximity to the granulocytes while few cells were seen to be devoid of contact with the GFP^+^ cells (Figure 2(a)). Most of the visualized hMSC-associated CTO signals occupied the perivascular region of the bone marrow, confirming the previously, published findings [30–32] (Figure 2(b)). However, the CTO-labeled hMSCs do not express GFP (Figure 2(b)). Upon close inspection, some CTO signals could be seen as intracellular inclusions within GFP^+^ LysM^+^ granulocytes, suggesting that cellular debris from infused CTO-labeled hMSCs were the source of these intracellular inclusion bodies found in LysM^+^ cells (Figure 2(c); white arrows). However, in the same imaging field we were also able to detect larger CTO^+^ cells that did not coexpress the GFP signal, suggesting that these may be intact, viable hMSCs that were infused a day earlier (Figure 2(c); yellow arrows). Thus, by subtracting the overlapping GFP signal from the CTO signal in the intravital imaging sequence, it was possible to distinguish intact hMSCs from GFP^+^ LysM^+^ granulocytes that had engulfed fluorescent hMSC debris. Our data strongly suggest that, contrary to recent reports, intact hMSCs do indeed survive past the lungs and can migrate to the bone marrow at least a day after *i.v.* administration [22]. 

**Figure 3:**
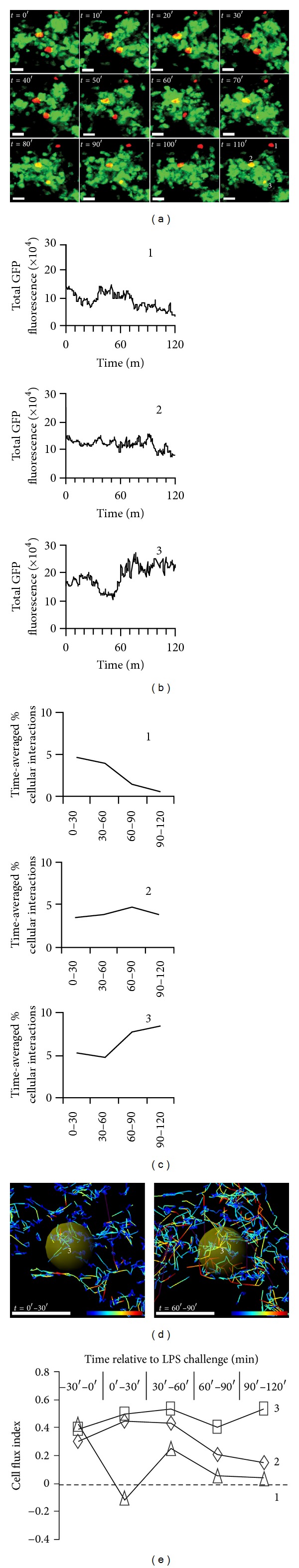
Time-lapse TPM images of LysM^+^ granulocytes interacting with hMSC after systemic LPS administration. CTO-labeled hMSCs (red) were injected *i.v.* into a *LysM* ^+/*GFP*^ recipient mouse containing LysM^+^ (green) granulocytes 20 hours prior to performing intravital TPM of the bone marrow through intact calvarium of the live recipient mouse. The recipient mouse received a 100 ng of LPS injection *i.v.* at relative time = 0 min (*t* = 0′). (a) Sequential TPM images from Supplemental Movie 1 (See Supplementary Materials available online at http://dx.doi.org/10.1155/2013/656839) displayed at 10-minute intervals for a total of 110 minutes (*t* = 110′) starting from the time of LPS injection (*t* = 0′), showing the accumulation of LysM^+^ GFP^+^ granulocytes surrounding 2 out of 3 hMSCs (red) that are clearly visible within the imaging field. Scale bar = 20 *μ*m. (b) Total fluorescence of GFP signal within a 20 *μ*m radius of each of the three hMSCs identified in (a) at *t* = 110′ over time. (c) Time-averaged percent of LysM^+^ GFP^+^ neutrophils within 20 *μ*m radius of the hMSC that formed a cell-cell contact with the said hMSCs after LPS challenge. The percent was normalized to the contact frequency at *t* = 0′. (d) Track analysis of cell number 3 in (a) *t* = 110′. Shown are migration tracks of surrounding LysM^+^ GFP^+^ granulocytes during 0 to 30 minutes (left panel) and 60 to 90 minutes (right panel) after LPS administration. Individual migration heat map tracks are color-coded based on the calculated instantaneous speed. Yellow sphere = 20 *μ*m radius around the center of hMSC number 3. Scale bar = 50 *μ*m. (e) Cell flux indices for 3 different hMSCs identified in (a) are shown, where all three hMSCs showed net increased neutrophil flux towards them after LPS administration, although hMSC number 3 exhibited heightened and sustained positive cellular flux. hMSC number 2 had an initial increase in positive flux, which reached homeostasis two hours after LPS injection. hMSC number 1 initially had a negative cellular flux due to local neutrophils being attracted to hMSCs number 2 and number 3. However, neutrophils from regions beyond the imaging field began to migrate towards the hMSC 30 to 60 minutes following LPS injection before the cellular traffic reached a steady-state level (cell flux index of ~ 0).

Armed with the technical capability to detect individual hMSCs and granulocytes in intact bone marrow of a live mouse, we applied time-resolved intravital TPM to visualize the response and interaction dynamics of granulocytes and hMSCs in the bone marrow of recipient mice undergoing active systemic LPS challenge (Figure 3). With the anesthetized, hMSC-infused *LysM* ^+/*GFP*^ mouse situated under the TPM imaging objective during a continuous sequential *xyz *imaging data acquisition, we injected 100 ng of *S. enterica* LPS *i.v*. and visualized the behavior of both hMSCs and GFP^+^ granulocytes (Supplemental Movie 1, See Supplementary Materials available online at http://dx.doi.org/10.1155/2013/656839). LysM^+^ cells interacted with the hMSCs only sporadically during both the 1-hour imaging before LPS injection (data not shown) and the first 30 minutes after LPS injection (Figure 3; *t* = 0′–30′). Starting around 30 minutes following LPS injection, we observed dramatic changes in granulocyte behavior, including increases in their instantaneous speed and apparent overall activity (Figure 3). By 50 minutes post-LPS administration, the granulocytes began to cluster (“swarm”) around some of the hMSCs (Figures 3(a) and 3(d); Supplemental Movie 1). We examined several parameters which together contribute to the observed neutrophil “swarming” behavior, namely: (I) total cellular accumulation (reflected as changes in total GFP fluorescence; Figure 3(b)); (II) frequency of neutrophil-hMSC contact (calculated as time-averaged % cellular interactions; Figure 3(c)); (III) migration speed and trajectory of neutrophils (Figure 3(d)); and (IV) flux of neutrophils coming towards and leaving from the neutrophil-hMSC cluster (enumerated as cell flux index; Figure 3(e)). The total GFP signals (as a reflection of total GFP^+^ granulocytes) around the tracked hMSCs were variable, with some decreasing (Figure 3(b), panel 1) or exhibiting transient fluctuation in signal intensity (Figure 3(b), panel 2), while other hMSCs experienced dramatic increases in GFP signal intensity over time following LPS injection (Figure 3(b), panel 3). In order to further demonstrate the dynamic recruitment, clustering, and direct cellular interactions between GFP^+^ cells and some hMSCs, we analyzed the time-averaged percentage of GFP^+^ cells that traversed to within a 20 *μ*m radius of the hMSCs. Some, but not all, of the hMSCs experienced increasing numbers of interactions with GFP^+^ granulocytes starting at 60–90 minutes after LPS treatment (Figures 3(c) and 3(d)). These dynamic data indicate that the observed hMSCs did not all encounter the same granulocyte “swarming” behavior at the same time or to the same extent, as some labeled hMSCs did not appear to participate in the “swarming” behavior during the entire imaging session. Our observation helps to explain the observed behavior of LysM^+^ cells showing that granulocyte clusters can form in different spots within the bone marrow at various times following LPS administration (Supplemental Movie 1). Some of the “swarming” locations occurred in areas without any observable labeled hMSCs and may presumably be caused by the presence of unlabeled, endogenous stromal cells or MSCs that were stimulated with the TLR4 agonist [33–37]. 

**Figure 4:**
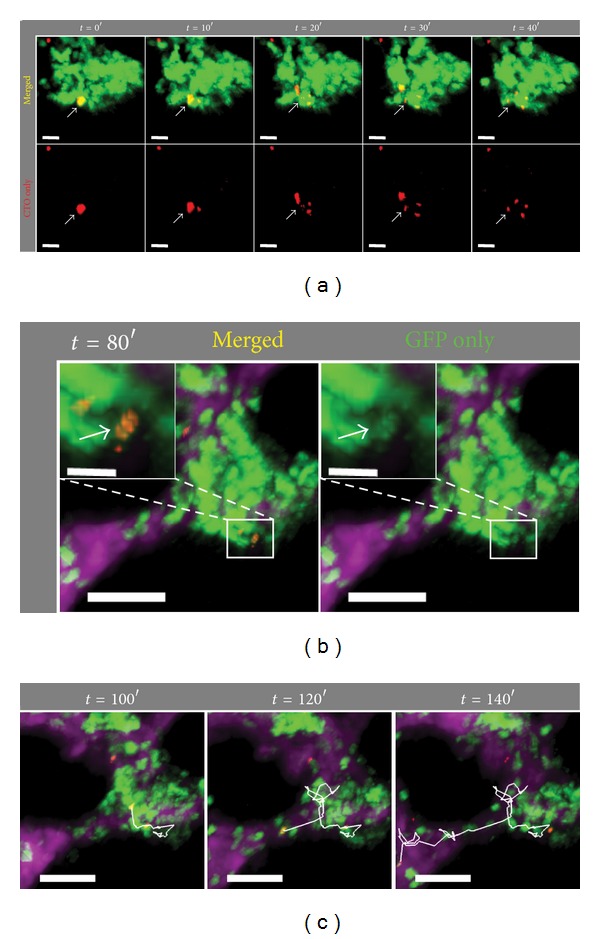
Cellular fragmentation of a dying hMSC followed by phagocytosis of migrating LysM^+^ GFP^+^ granulocyte engulfing hMSC debris. (a) Intravital TPM images of a different area of the calvarium bone marrow from Figures 2 and 3, demonstrating the dramatic fragmentation of the CTO-labeled (red) hMSC cell body (white arrow) into at least 4 pieces over a period of 40 minutes (*t* = 0′ corresponds to 20 minutes prior to LPS injection). Scale bar = 20 *μ*m. (b) A LysM^+^GFP^ dim^ cell containing phagocytosed CTO-labeled hMSC debris is shown to demonstrate colocalization of GFP (green) and CTO (red) signals (inset: zoomed view at *t* = 80′ with (left; “merged”) and without (right; “GFP only”) CTO signal). (c) A LysM^+^ GFP^+^ CTO^+^ cell exhibited a highly motile behavior over a long distance in the bone marrow (white tracks) over a 60-minute imaging period. Scale bar outset = 50 *μ*m; inset scale bar = 10 *μ*m.

Understanding the fate of infused hMSCs is of particular clinical relevance as their persistence and elimination *in vivo* may correlate with their clinical efficacy. As shown in Figure 2, LysM^+^ granulocytes were able to engulf CTO-labeled hMSC fragments that were distinguishable from intact, viable hMSCs. To this end, we were able to use the dynamic intravital TPM to visualize the active process of dying hMSC fragmentation and subsequent distribution and digestion of hMSC cell fragments by multiple LysM^+^ cells in the bone marrow (Figure 4; Supplemental Movie 2). Over a 40-minute period, we observed an hMSC digested into multiple large fragments (Figure 4(a)). These fragments were taken up by multiple LysM^+^ cells (Figure 4(b)). At least one of the CTO^+^ GFP^+^ cells was seen to migrate away from the cluster into the blood vessel (Figure 4(c)). The fluorescence intensity of the CTO-containing GFP^+^ cell appeared dimmer compared with surrounding bright neutrophils (Figure 4(b), *t* = 80′ inset), suggesting that the migrating LysM^+^GFP^ dim^ cell may represent a monocytic cell [38, 39]. It is interesting to note that the observed fragmentation process of hMSC began before any LPS was administered to the animal, indicating that a portion of infused hMSCs were actively being eliminated even before onset of inflammation. Additionally, the intact hMSCs initially observed in the beginning of the imaging session (one hour prior to LPS administration) were still visibly intact during the entire two-hour imaging tracking following LPS injection throughout the neutrophil swarming behavior. Analyzing all available imaging datasets, we observed a total of 151 CTO^+^ events during the one-hour imaging session prior to LPS administration, with 18 CTO^+^ signals comprised of intact hMSC cells (11.9%). During the two-hour data acquisition following LPS administration, we observed a total of 143 CTO^+^ events, with 14 CTO^+^ signals generated from intact hMSCs (9.8%). There was no statistically significant difference (*P* = 0.36) between the percentage of intact CTO^+^ hMSCs before and after LPS challenge, indicating that, at least within the limits of the 3-hour imaging session, LPS administration did not cause an accelerated destruction of hMSCs in the marrow. 

## 4. Discussion and Conclusion

There has been much debate about how therapeutically administered hMSCs exert their immune-modulatory function *in vivo *(i.e., systemic versus local effect). In rodents, hMSCs have been shown to confer therapeutic effects on the outcome of various disease models including systemic lupus erythematosis (SLE) and sepsis [40–42]. An emerging paradigm indicates that hMSCs can exhibit both proinflammatory (type 1) and anti-inflammatory (type 2) responses *in vivo *depending on the specific stimuli encountered by the hMSCs [35]. In particular, this modulation of hMSC function was shown to occur during stimulation with Toll-like receptor (TLR) agonists [33, 35, 37]. In response to stimulation with the TLR4 agonist LPS, bone marrow MSCs upregulate inflammatory cytokines such as MCP-1 [36] and IL-8 [34] and can affect mobilization of granulocytes. All of the information regarding the role(s) of MSCs in inflammation, however, has come from indirect methods involving bulk cell analysis with virtually no information on the cellular interaction dynamics between granulocytes and MSCs during inflammatory responses. 

In this study, we tracked the fate of infused hMSCs in the bone marrow of immune competent mice and observed their dynamic interactions with host bone-marrow-resident LysM^+^ neutrophils and monocytes before and after systemic LPS administration. Our *in situ *single-cell imaging data directly refutes a prior published report claiming that intravenously administered mouse MSCs were unable to reach tissues other than the liver, lung, and the spleen, and that the biological effect of hMSCs *in vivo* is therefore likely due to systemic effects [22]. As observed in our dynamic imaging studies, live hMSCs were able to traffic to the bone marrow, survive in a xenograft environment with an intact host immune system, and interact with surrounding neutrophils following systemic challenge with a TLR agonist. At the same time, we also observed the destruction and subsequent phagocytosis of hMSCs by host immune cells. 

Of particular interest is our finding that mouse granulocytes exhibit a highly dynamic “swarming” behavior around hMSCs in response to LPS challenge and that hMSCs remain essentially intact during such an active host innate immune response. The observed granulocyte behavior was similar to that described in peripheral organs such as the liver during infectious processes [38], suggesting that the clustering and dynamic behavior was a general phenomenon of the granulocytes *in vivo*. The hMSCs have been shown to directly respond to TLR stimulation [33–37] and release chemokines MCP-1 and IL-8 for neutrophil recruitment [34, 36]; therefore, it is possible that the bone marrow neutrophils were actively responding to local chemokines released by the hMSCs following LPS stimulation. Alternatively, the neutrophils may respond directly to LPS [43] or signals that are released by other cell types in the bone marrow as a result of TLR stimulation, and that both hMSCs and granulocytes nest in the same niche where the signal source resides or the same anatomical sites where exiting and entering cells colocalize in the bone marrow. Additional experiments are needed to interrogate the roles of various nonfluorescent stromal and marrow-derived elements that appeared “dark” or were not labeled in our imaging background during this dynamic inflammatory interplay between hMSCs and host cells and the destruction of hMSCs in the xenograft environment as witnessed in the current study. In addition, future investigations are needed to address how *in vivo *interactions with the hMSCs affect functional and behavioral changes in neutrophils and other host immune cells. 

A limitation in our current study is the lack of lineage-specific fluorescent markers to differentiate the GFP^+^ responding cells that were actively engaging hMSCs. While >95% of GFP^+^ cells in our mouse model express Gr1^+^/CD11b^+^/GFP^ bright^ markers consistent with neutrophils [38, 39], we cannot determine with certainty the true identity of the imaged GFP^ dim^ cells as belonging to either the monocyte/macrophage or neutrophil lineages (Figure 4(b), inset). Multicolor lineage-specific fluorescent reporters will be required to further distinguish the responses these cell types of hMSCs and LPS challenge. Another limitation of this study is that our description of the hMSC behavior with neutrophils was restricted to the calvarium bone marrow and not other marrow cavities such as that found in the femurs and other long bones. As intravital imaging of the latter would require additional manipulations such as removal of bone and muscles, as well as mechanical thinning of the cortical bone, these procedures may potentially introduce additional trauma to the underlying hMSC-neutrophil biology. For this reason we purposely restricted our observation to the calvarium marrow in order to minimize tissue damage and inflammation. 

We were also intrigued by the variable interaction kinetics between neutrophils and different hMSCs in the bone marrow (Figure 3) with some of the infused hMSCs undergoing apoptosis while others remained intact (Figure 4). Interestingly, the rate of hMSC destruction and subsequent phagocytosis by LysM^+^ cells was not exacerbated by LPS stimulation. The variable interaction kinetics may be due to heterogeneous states of differentiation and maturation within the infused hMSC population incurred either during the *in vitro* culture process or as a result of different local signals received by hMSCs within different microniches in the bone marrow. Another, though less likely, possibility is cellular contaminant in the infused cell cohort that was not MSCs. In the future, a possible way to address this issue will be to construct marker-specific fluorescent reporters in the infused hMSCs rather than employing nonspecific, pan-cell fluorescent dyes. In either scenario, it is important to understand how these cells behave *in vivo* as they represent the actual cellular products that are currently being infused into patients who are enrolled in a variety of active clinical trials. Furthermore, as recent data support the notion that neutrophils are active participants in a variety of adaptive immune responses [44–47], it would be extremely interesting to study the associated functional changes in the hMSC debris-containing neutrophils in a variety of inflammatory or pathological settings, and whether phagocytosis of hMSC cellular components may represent a potential mechanism by which hMSCs exert their immune-modulatory effect *in vivo*. Together, our current study demonstrated the value and utility of intravital TPM approach in furthering our limited understanding of the biology and *in situ *cellular mechanisms by which hMSCs traffic to and exert therapeutic and immune-modulatory effects upon host tissues.

## Supplementary Material

Dynamic interactions were visualized between hMSCs and surrounding LysM^*+*^ GFP^*+*^ granulocytes in the bone marrow following systemic LPS administration (Supplemental Movies 1 and 2). A total of 8×10^6^ CTO-labeled hMSCs (red) were injected *i.v.* into a *LysM^*+*/GFP^* recipient mouse containing LysM^*+*^ (green) neutrophils and monocytes 20 hours prior to the imaging experiment. Intravital TPM was then performed through the intact calvarium of the live recipient mouse before and after *i.v.* injection of 100ng LPS. Supplemental Movie 1 shows the cellular interactions before and after LPS injection as characterized in Figure 3. LPS was injected at time = 60 mins of the movie which corresponds to t=0' of Figure 3. Supplemental Movie 2 shows the cellular fragmentation of a hMSC by LysM^*+*^ GFP^*+*^ granulocytes followed by active migration of the CTO-containing, LysM^*+*^ GFP^*+*^ phagocytes as characterized in Figure 4. This movie is taken from another imaging area of the same mouse calvarium bone marrow as shown in Supplemental Movie 1. In this movie, a CTO^*+*^ hMSC was seen in close juxtaposition to surrounding LysM^*+*^ GFP^*+*^ granulocyte before the start of LPS administration. Over the course of 40 minutes, the hMSC was observed to break apart into multiple pieces of cellular debris, each of which was phagocytosed by a surrounding LysM^*+*^ GFP^*+*^ granulocyte. A GFP^*+*^ cell containing engulfed CTO-cellular debris was subsequently seen traversing the bone marrow cavity at a high rate of speed over a 60-min period.
